# Multi-resolution visual Mamba with multi-directional selective mechanism for retinal disease detection

**DOI:** 10.3389/fcell.2024.1484880

**Published:** 2024-10-11

**Authors:** Qiankun Zuo, Zhengkun Shi, Bo Liu, Na Ping, Jiangtao Wang, Xi Cheng, Kexin Zhang, Jia Guo, Yixian Wu, Jin Hong

**Affiliations:** ^1^ Hubei Key Laboratory of Digital Finance Innovation, Hubei University of Economics, Wuhan, China; ^2^ School of Information Engineering, Hubei University of Economics, Wuhan, China; ^3^ Hubei Internet Finance Information Engineering Technology Research Center, Hubei University of Economics, Wuhan, China; ^4^ School of Mathematics and Computer Science, Nanchang University, Nanchang, China; ^5^ School of Mechanical Engineering, Beijing Institute of Petrochemical Technology, Beijing, China; ^6^ School of Information Engineering, Nanchang University, Nanchang, China

**Keywords:** retinal disease detection, state-space model, global–local feature, multi-scale fusion, multi-directional selective learning

## Abstract

**Introduction:**

Retinal diseases significantly impact patients’ quality of life and increase social medical costs. Optical coherence tomography (OCT) offers high-resolution imaging for precise detection and monitoring of these conditions. While deep learning techniques have been employed to extract features from OCT images for classification, convolutional neural networks (CNNs) often fail to capture global context due to their focus on local receptive fields. Transformer-based methods, on the other hand, suffer from quadratic complexity when handling long-range dependencies.

**Methods:**

To overcome these limitations, we introduce the Multi-Resolution Visual Mamba (MRVM) model, which addresses long-range dependencies with linear computational complexity for OCT image classification. The MRVM model initially employs convolution to extract local features and subsequently utilizes the retinal Mamba to capture global dependencies. By integrating multi-scale global features, the MRVM enhances classification accuracy and overall performance. Additionally, the multi-directional selection mechanism (MSM) within the retinal Mamba improves feature extraction by concentrating on various directions, thereby better capturing complex, orientation-specific retinal patterns.

**Results:**

Experimental results demonstrate that the MRVM model excels in differentiating retinal images with various lesions, achieving superior detection accuracy compared to traditional methods, with overall accuracies of 98.98\% and 96.21\% on two public datasets, respectively.

**Discussion:**

This approach offers a novel perspective for accurately identifying retinal diseases and could contribute to the development of more robust artificial intelligence algorithms and recognition systems for medical image-assisted diagnosis.

## 1 Introduction

The human body relies on the eyes to perceive external information. However, the eyes are easily damaged because of prolonged screen exposure, resulting in frequent vision problems and serious interference with daily life Rauchman et al. (2022). In today’s society, the popularity of electronic devices such as mobile phones and computers makes it almost impossible to work and study without using electronic screens, which undoubtedly poses a direct challenge to vision. Long-term immersion in front of electronic screens often leads to varying degrees of vision damage Lanzani et al. (2024). Due to the large population base and uneven distribution of medical resources, not everyone can receive high-quality medical diagnosis and treatment in time, which increases the risk of delayed illness and makes some patients miss the best time for treatment. According to the World Health Organization, approximately 2.2 billion people in the world have vision problems caused by eye diseases Bashshur and Ross (2020). It is particularly noteworthy that nearly half of these vision impairments could have been avoided or recovered through effective preventive measures or early and timely intervention. Therefore, in the field of clinical research, early detection and accurate diagnosis of eye diseases Xu et al. (2022); Wan et al. (2023b); Wan et al. (2024b) are particularly important. Accurate diagnosis of eye diseases can not only reduce avoidable vision loss, but also improve the quality of patients’ life.

**FIGURE 1:**
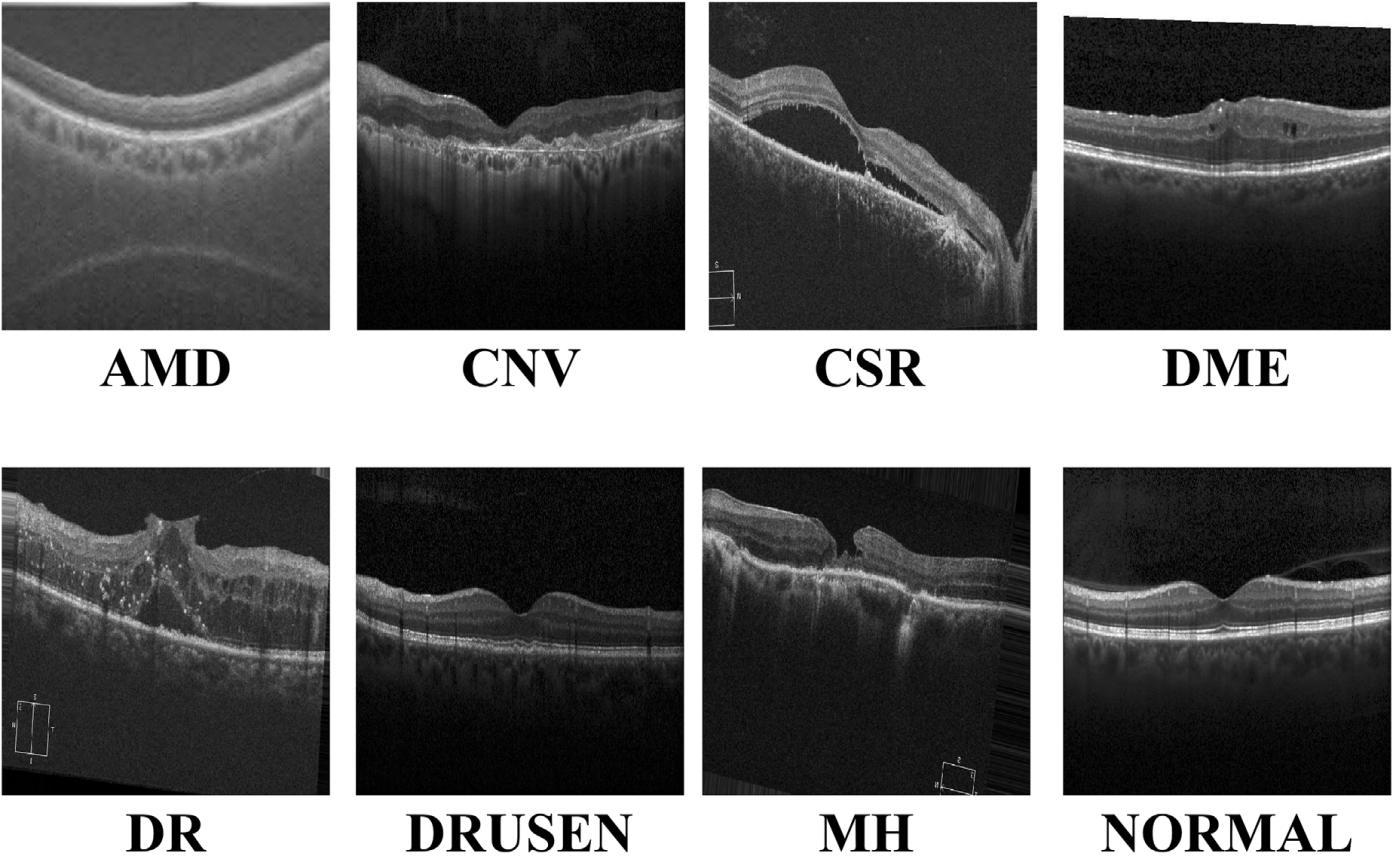
Visualization of the eight retinal diseases.

With the continuous advancements in optimal theory and technology Wan et al. (2023a); Wan et al. (2024a); Ji et al. (2024), optical coherence tomography (OCT) technology has emerged and rapidly penetrated into the medical field Bouma et al. (2022). OCT has significant advantages such as high resolution, efficient detection, and non-invasiveness. It can be used for the detection and diagnosis of retinopathy and has now become an indispensable routine method in eye examinations Xu et al. (2023). Figure 1 shows eight examples of retinal disease, namely, age-related macular degeneration (AMD), choroidal neovascularization (CNV), central serous chorioretinopathy (CSR), diabetic macular edema (DME), macular hole (MH), Drusen, diabetic retinopathy (DR), and normal. However, due to hardware and equipment factors, OCT images are often mixed with unavoidable noise during the imaging process, which undoubtedly increases the complexity and challenge of diagnosis for doctors. Moreover, OCT is a grayscale imaging technique. Since the characteristics of small lesions are not clear enough at the grayscale level, these subtle changes are often difficult to detect, which increases the risk of missed diagnosis by doctors. At the same time, although the number of patients with retinal eye diseases increases year by year, the number of doctors with professional diagnostic capabilities is relatively scarce. This contradiction is becoming increasingly prominent, making it difficult to effectively meet the diagnosis and treatment needs of a large patient population Daich Varela et al. (2023). This technology can assist doctors in accurately assessing patients’ conditions, effectively reducing doctors’ workload, while improving the accuracy of eye disease screening and diagnosis. It has far-reaching significance for optimizing the allocation of medical resources and improving the quality of medical services.

In the field of medical image processing, convolutional neural networks (CNNs) have performed well in medical image segmentation Li et al. (2024); Hong et al. (2022b); Zhang et al. (2023), image generation You et al. (2022); You et al. (2024), and image classification Yu et al. (2022); Zong et al. (2024); Zuo et al. (2023a). By stacking multiple layers of convolution and pooling layers, CNNs can effectively extract complex features and subtle lesions in images Hong et al. (2022a), such as microaneurysms and exudates, which are key signs of diseases such as diabetic retinopathy. Combined with fully connected layers for feature integration and classification, CNN models can accurately distinguish different types of retinal diseases, providing ophthalmologists with fast and objective preliminary diagnostic references, thereby improving the diagnostic efficiency and accuracy and speeding up patient treatment. However, CNN models have difficulty modeling long-distance dependencies in images and are sensitive to position translation, which limits their application in certain complex retinal disease classification tasks.

Due to its remarkable work in natural language processing, the transformer network is now gradually entering the field of medical image computing Zuo et al. (2024); Zuo et al. (2023b), bringing improvements in performance of the task of retinal disease image classification Parvaiz et al. (2023). Due to the unique self-attention mechanism, the transformer-based network is able to deeply analyze the complex relationship between each pixel and other pixels in the image, thereby capturing small but important pathological features in retinal disease images, such as subtle vascular abnormalities and exudate distribution. This global information integration capability enables the transformer network to more accurately identify different types of retinal diseases during the classification process, providing ophthalmologists with a more reliable and timely diagnostic basis. Since the network does not consider the spatial locality of the image, it may not capture detailed features as finely as CNNs when processing high-resolution medical images and requires larger data sets and computing resources to train, all of which limit the application scenarios of transformer-based models in medical image diagnosis.

Recently, the Mamba network, an innovative deep learning architecture, has excelled in long-distance relationship modeling Gu and Dao (2023); Zhu et al. (2024). Through its unique selection state mechanism, it effectively captures the spatial dependencies between distant regions in an image and ignores noise interference, thereby improving the learning efficiency and prediction accuracy of the model. Inspired by the above observations, we combined the CNN and Mamba networks and proposed the multi-resolution visual Mamba (MRVM) model for OCT image classification. The MRVM model first extracts local features from OCT images using convolution and then captures global long-range dependencies through the retinal Mamba. Next, by integrating multi-scale global features, the model enhances the classification accuracy and overall performance. The multi-directional selection mechanism (MSM) within the retinal Mamba improves feature extraction by focusing on various directions, thereby boosting the model’s ability to detect complex, orientation-specific retinal patterns. Finally, the fused multi-scale features are sent to the classifier to discriminate disease-related OCT images. The proposed model has the potential to accurately detect retinal diseases and can be extended to other medical image classifications. The main contributions of this work are summarized as follows.

• The proposed MRVM model first extracts local features of OCT images through the convolution module and then extracts global long-range dependent features through the retinal Mamba, significantly improving the performance of image analysis and recognition tasks.

• We devised the MSM in the retinal Mamba to enhance feature extraction by focusing on multiple directions of the local receptive feature map. This enables the model to more effectively capture complex, orientation-specific patterns in retinal images, improving the performance of image classification and retinal disease detection.

• By fusing multi-scale global features, it can capture detailed lesion characteristics of retinal images at different scales, further improving the performance of OCT image classification and making the model more robust and accurate.

The subsequent sections of this work are structured as follows: In Section 2, we review the literature on retinal disease detection. We detail the innovative MRVM model in Section 3 to introduce a novel approach for detecting retinal disease using OCT images. Subsequently, Sections 4 and 5 present the experimental setup alongside comparative prediction outcomes utilizing alternative methods. Lastly, Section 6 delves into the credibility of this work and provides concise key findings.

## 2 Related works

The classification performance of retinal OCT images is also constantly improving with the advancement of artificial intelligence. These improved methods mainly focus on local feature learning and global feature learning.

The first approach focuses on local lesion characteristics. It deeply analyzes the key lesion signs in the image, such as changes in the vascular morphology, edema of the optic disc, and abnormal manifestations of the macular area, and accurately captures the specific characteristics of these lesions to achieve accurate classification of the retinal diseases. Rong et al. (2018) proposed a CNN-based automatic classification method to effectively classify OCT images through image denoising, mask extraction, and proxy image generation. This CNN-based method performs well in evaluation on different databases. Alqudah, (2020) developed a more powerful CNN-based model to classify five types of retinal diseases (including AMD, CNV, DME, Drusen, and normal) with an overall accuracy of 95.3%. Karthik and Mahadevappa (2023) replaced the residual connection with the contrast of derivatives in the standard ResNet model. Experimental results on the two public OCT datasets show at least 1% improvement in the accuracy estimation. To reduce the model size, Sunija et al. (2021) designed only six convolutional blocks with downsampling and weight sharing mechanisms to classify four-label OCT images. Compared with the existing ResNet-50 model, it uses 6.9% of the learnable parameters but has a better classification performance. Considering the previous methods may ignore useful discriminative information at different scales, Wang and Wang (2019) designed a novel CNN-based method to automatically detect AME and AMD, which shows good classification performance in cross-dataset adaptability. In addition, Das et al. (2021) proposed a deep multi-scale fusion convolutional neural network (DMF-CNN) to extract and fuse different scale features for AMD/DME/normal classification. The multi-label classification results show excellent performance and good versatility on the UCSD and NEH datasets.

The second approach is modeling the global diseased areas, which focuses on the overall information of the image, comprehensively considers multiple visual elements and structural features in the image, and does not need to identify specific lesions separately but directly performs intelligent analysis on the entire image so as to determine the label of retinal diseases from a global perspective. Yu et al. (2021) applied the vision transformer (VIT) to the task of retinal disease classification. Their framework outperforms CNN models on two publicly funded image datasets. Shen et al. (2023) incorporated the clinical prior knowledge to guide the transformer-based network for retinal disease prediction and achieved superior classification and good generality on the public nAMD dataset. Hammou et al. (2023) used the pre-trained state-of-the-art models as the prior knowledge and fine-tuned these models to classify OCT videos. This method has potential application in the real-time diagnosis of retinal diseases. To improve the accuracy and interpretability of these classification models, He et al. (2023) proposed a transformer-based model with Swin-poly strategy to classify retinal OCT images. They achieved state-of-the-art performance on the OCT2017 dataset, which is superior to that of both vision transformer (VIT) and convolutional neural network approaches. A similar work is presented in Playout et al. (2022). Wen et al. (2022) combined the transformer and CNN to train this hybrid model for ophthalmic disease classification. This model extracts both local and global contexts for lesion area extraction and understanding with considerable accuracy improvement. In addition, they Laouarem et al. (2024) designed a hybrid model to classify seven retinal diseases by combining visual transformers and CNN. They extracted multi-scale local features from OCT images by a hierarchical CNN and achieved good results on three public datasets. Hemalakshmi et al. (2024) proposed a SqueezeNet-Vit model to extract local and global features for more accurate OCT classification.

## 3 Methods

**FIGURE 2:**
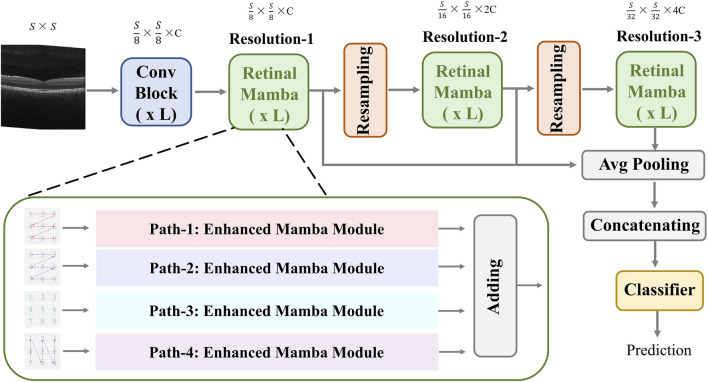
Architecture of the proposed MVRM model, consisting of the Conv block, retinal Mamba module, sampling module, and classifier. The input is a two-dimensional image, and the output is a vector representing the retinal disease label.

The proposed MVRM model is illustrated in Figure 2. The input is an image with the size S×S , and the output is the retinal disease label. There are three main blocks: the convolutional block, the retinal Mamba block, and the classifier block. The convolutional block is used to extract local structures buried in the image by using local receptive fields and parameter sharing. The local receptive field allows the convolution kernel to focus on only a small area, thereby capturing local features. The retinal Mamba focuses on the long-range dependencies and mines the overall lesion area association in OCT images. Through the resampling modules, the three retinal Mamba modules can generate multi-scale global–local features for capturing the characteristics of the lesion area from all directions. By cleverly integrating global features and local features, the proposed model not only fully retains disease-related global information but also significantly enhances its ability to keenly capture local subtle differences. This fusion strategy effectively improves the accuracy and robustness of classification tasks. Furthermore, by using the category loss function to optimize and calculate these fused multi-scale features, the model can generate more refined and representative representations for each retinal disease category. These representations accurately reflect the core characteristics of retinal diseases and can be used for analysis and decision-making on other downstream tasks. The details of these blocks are described in the following sections.

### 3.1 Convolutional block

In the convolution module, we designed three residual layers, and the output sizes of these three residual layers are as follows: $\left(S/2\right)\times \left(S/2\right)\times C_{1}$ , $\left(S/4\right)\times \left(S/4\right)\times C_{2}$ , and $\left(S/8\right)\times \left(S/8\right)\times C_{3}$ . Adjacent residual layers are connected with 1 × 1 convolution kernels with a sliding step of 2. After the third residual layer, a 1 × 1 convolution kernel is used to change the number of channels from $C_{3}$ to C . The input image size is S×S , and the output size is (S/8)×(S/8)×C . The calculation formula can be expressed as follows:

$$I_{1}=Residual\left(I_{0}\right).$$ (1)

$$I_{2}=Residual\left(I_{1}\right).$$ (2)

$$I_{3}=Conv_{1\times 1}\left(Residual\left(I_{2}\right)\right).$$ (3)

$$Residual=\left(Conv_{3\times 3},BN,ReLU,AvgPool,Conv_{1\times 1},BN,ReLU,AvgPool\right)+shortcut\left(Conv_{1\times 1}\right).$$ (4)

where, Equations 1–3 are based on the Equation 4. In Equation 4, it contains 2 sub-convolution layers. The first sub-convolution layer contains a 3 × 3 convolution (Conv) kernel with a step size of 2, a batch normalization layer (BN), a ReLU activation layer, and an average pooling layer (AvgPool); the second sub-convolution layer contains a 3 × 3 convolution kernel with a step size of 1, a normalization layer, a ReLu activation layer, and a flat pooling layer.

### 3.2 Retinal Mamba

This module extracts global disease-related patterns by selectively modeling different parts of the OCT image. To capture multi-scale patterns, we designed two resampling modules to obtain multi-resolution feature maps and utilize the retinal Mamba (RM) to learn the global lesion area relations from multi-scale perspectives. The resampling module between retinal Mamba modules consists of a batch-normalized 3×3 CNN layer with a stride of 2 to halve the image resolution and double the channel dimension. The multi-scale feature maps can be computed by the following formula:

$$R_{1}=RM\left(I_{3}\right).$$ (5)

$$R_{2}=Resampling\left(RM\left(R_{1}\right)\right).$$ (6)

$$R_{3}=Resampling\left(RM\left(R_{2}\right)\right),$$ (7)

where $R_{1}$ , $R_{2}$ , and $R_{3}$ are the output of Equations 5–7, representing feature maps at three different multi-resolutions. The feature map sizes are S/8×S/8×C , S/16×S/16×2C , and S/32×S/32×4C , respectively. Next, we use the average pooling to normalize the three multi-resolution maps and concatenate these maps to fuse multi-scale features. The fused feature $R_{f}$ can be expressed by the following:

$$R_{f}=AvgPool\left(R_{1}\right)\;\|\;AvgPool\left(R_{2}\right)\;\|\;AvgPool\left(R_{3}\right).$$ (8)

The fused feature $R_{f}$ in Equation 8 has the size 1×7C .

#### 3.2.1 Enhanced Mamba

**FIGURE 3:**
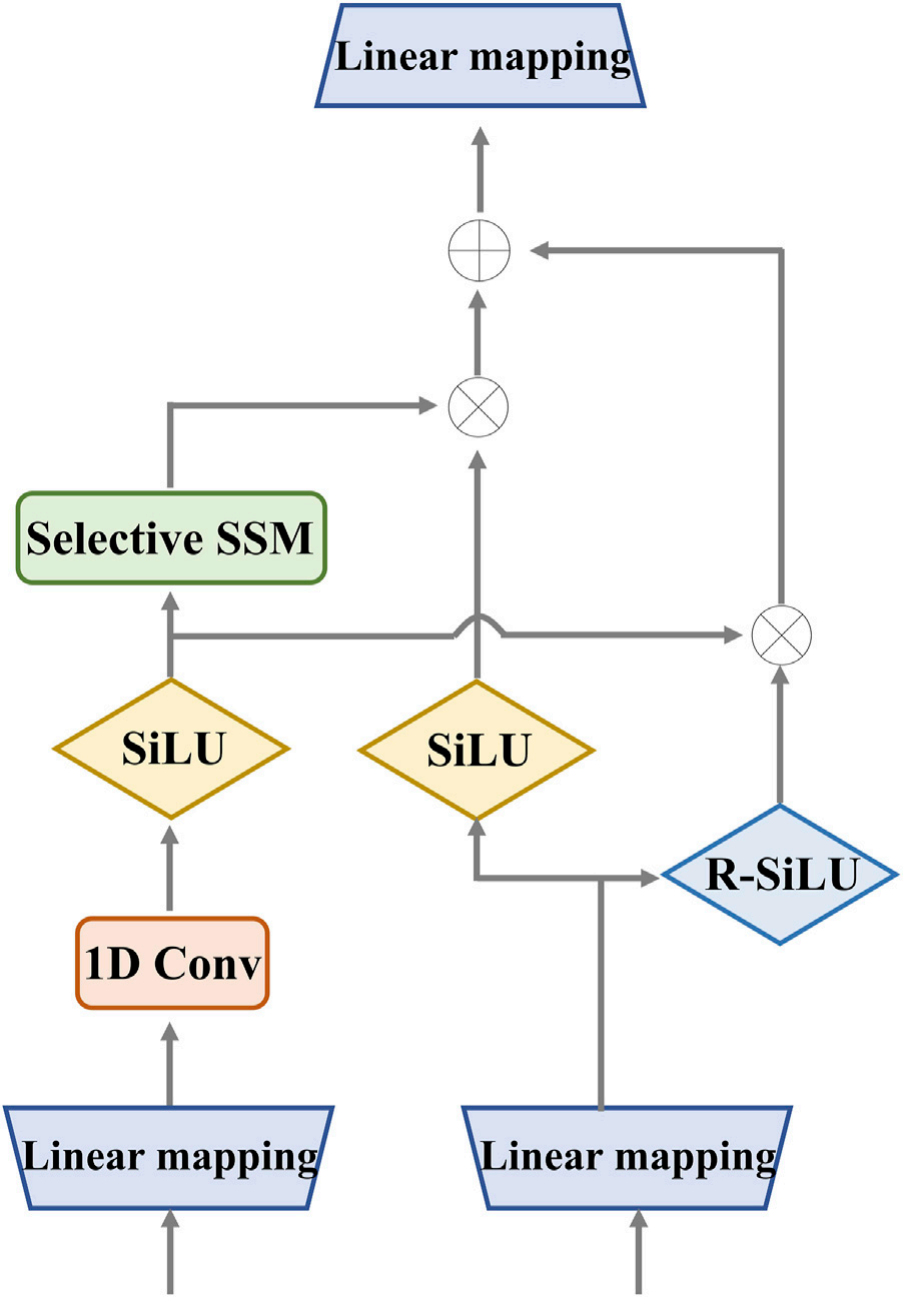
Detailed structure of the enhanced Mamba. It utilizes two gates to capture sequence dependencies for global complementary information. The input and output have the same dimension.

In the retinal Mamba, four paths are used to extract different direction features from the retinal OCT image. Considering the rich pattern correlations in different directions of time series and the complexity of spatial location dependencies, the output of each enhanced Mamba is added to fuse different directional features. The structure of each enhanced Mamba is shown in Figure 3.

We designed the enhanced Mamba with two pathways. The first pathway leverages a linear mapping (LM), a 1-D convolutional module, and a selective state-space model (SSM) to learn long-range sequence dependencies. The selective SSM can memorize long-term historical information in the HIPPO matrix. The second pathway generates two gates: the sigmoid-weighted linear unit (SiLU) and the reversed SiLU (R-SiLU). The SiLU gate processes the longer-term historical context, and the R-SiLU gate filters the complementary historical information to more comprehensively preserve the valuable long-term information. This designed enhanced Mamba facilitates a more nuanced and effective handling of long-term sequence modeling tasks. The computation process is illustrated in the Algorithm 1.

Algorithm 1 Computation process of enhanced Mamba.

**Input:** $Batch\left(R_{0}\right)$ : (b,l,d)

**Output:** $Batch\left(R_{1}\right)$ : (b,l,d)

 1: $x_{11}$ : (b,l,d) $\leftarrow \;LM_{11}$ ( $R_{0}$ )
 2: $x_{21}$ : (b,l,d) $\leftarrow \;LM_{21}$ ( $R_{0}$ )
 3: $x_{12}$ : (b,l,d) $\leftarrow \;SiLU\left(Conv1D\left(x_{11}\right)\right)$

 4: A : (d,q) $\leftarrow \;Parameter_{A}$

 6: C : (b,l,q) $\leftarrow \;LM_{C}\left(x_{12}\right)$

 7: Δ : (b,l,d) $\leftarrow \;log\left(1+exp\left(LM_{\Delta }\left(x_{12}\right)\right)\right)+Parameter_{\Delta }$

 8: $\bar{A},\bar{B}$ : (b,l,d,q) ← discretize( Δ , A , B )
 9: $y_{1}$ : (b,l,d) $\leftarrow \;SSM\left(\bar{A},\bar{B},C\right)$ ( $x_{12}$ )
 10: $y_{2}$ : (b,l,d) $\leftarrow \;y_{1}\cdot SiLU\left(x_{21}\right)+x_{12}\cdot \left(1-\sigma \left(x_{21}\right)\right)$

 11: $R_{1}$ : (b,l,d) $\leftarrow \;LM_{y_{2}}\left(y_{2}\right)$

 12: Return $R_{1}$

#### 3.2.2 Selective state-space model

**FIGURE 4:**
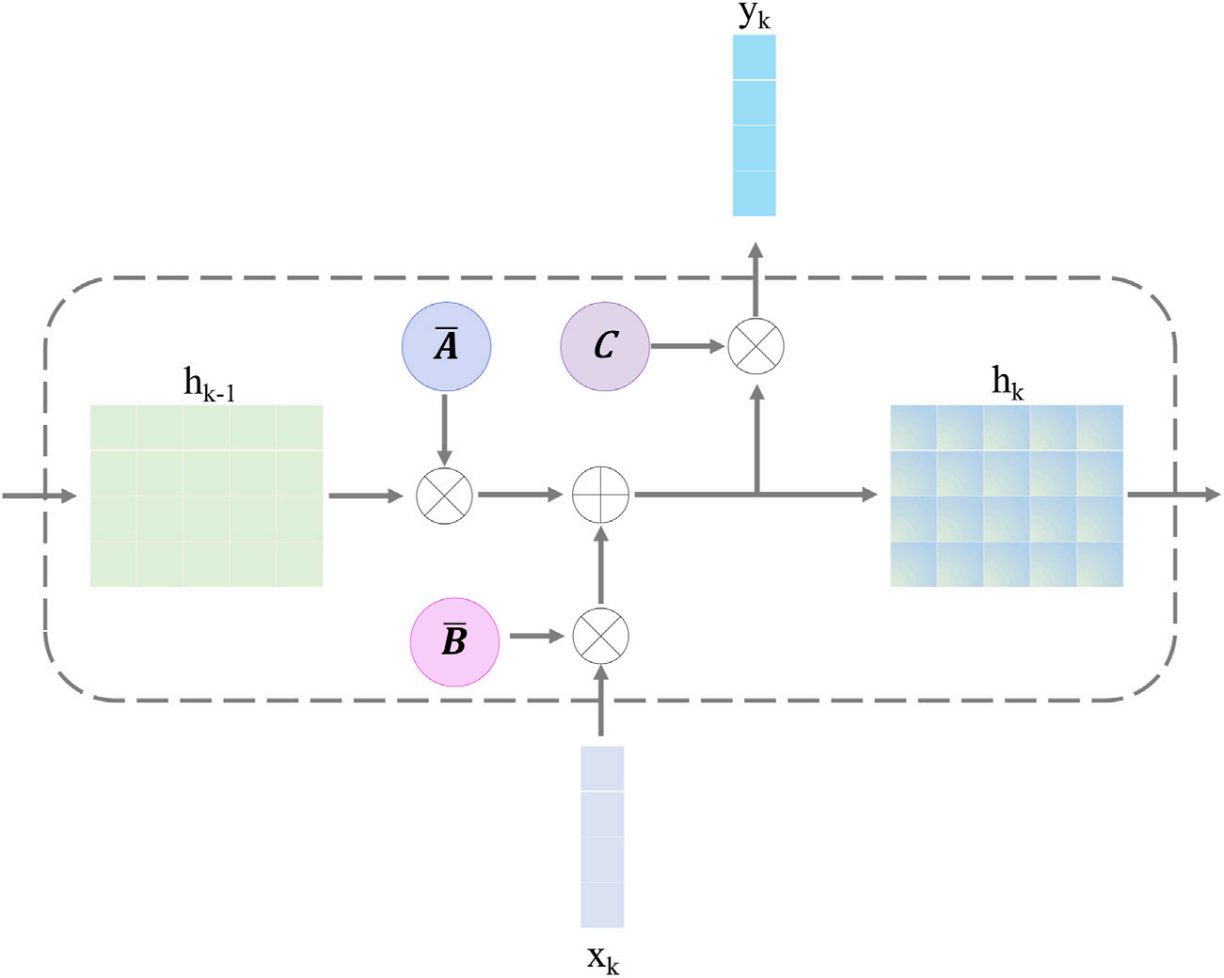
Structure of the selective state-space model.

The selective SSM can help the retinal Mamba to capture global dependencies in OCT images, capturing rich semantic disease-related information. The structure of the selective SSM is shown in Figure 4; it is a discretized version of the SSM, where the input is $x_{k}$ and the output is $y_{k}$ . Both of them are the features at the k -th time point. For the continuous condition, we map the one-dimensional sequence $x\left(t\right)\in R^{C}$ to the output sequence $y\left(t\right)\in R^{C}$ through latent historical representation h(t) . The continuous SSM is expressed as follows:

$$h\left(t\right)=Ah\left(t-1\right)+Bx\left(t\right),$$ (9)

$$y\left(t\right)=Ch\left(t\right).$$ (10)

Here, $A\in R^{C\times C}$ represents the state matrix, which memorizes the history information of latent representations. B and C project the input sequence and the latent representation into the output sequence. The problem of Equations 9, 10 lies in the unsuitable adaptation for deep learning. To solve this problem, we discretize it by introducing the time-scale factor Δ . The projection matrix B and the state matrix A can be transformed into $\bar{B}$ and $\bar{A}$ , respectively. The zero-order hold strategy is used to complete this task:

$$\bar{A}=exp\left(\Delta A\right),$$ (11)

$$\bar{B}={\left(\Delta A\right)}^{-1}\left(\bar{A}-I\right)\cdot \Delta B.$$ (12)

After discretizing with the step size Δ in Equations 11, 12, the SSM is defined with Equations 13, 14:

$$h_{k}=\bar{A}h_{k-1}+\bar{B}x\left(t\right),$$ (13)

$$y_{k}=Ch_{k}.$$ (14)

Finally, we employ a convolution operation for convenient optimization of the proposed model. The SSM computation is expressed as follows:

$$\bar{K}=\left(C\bar{B},C\bar{AB},\ldots ,C{\bar{A}}^{l-1}\bar{B}\right).$$ (15)

$$y=x*\bar{K},$$ (16)

where, in Equation 15, $\bar{K}$ indicates a dynamic convolutional kernel, and l denotes the sequence length. In Equation 16, x and y are matrices that share the same size l×d .

### 3.3 Classifier

The classifier is a five-layer perceptron network, including the three hidden layers. The input layer receives the fused feature $R_{f}\in R^{1\times 7C}$ . The three hidden layers have 5C , 3C , and C neurons, respectively. The output layer contains m neurons corresponding to retinal disease labels, and a softmax activation function is used to convert the output into a probability distribution, representing the predicted probability of each category. This network is trained using a back-propagation algorithm, adjusting weights and biases to reduce the error between the predicted category and the actual category. During the training process, the model learns to map the features of the input data to the corresponding category labels, thereby achieving classification. We utilized the cross-entropy objective to optimize the proposed MVRM model.

$$Y^{\prime }=classifier\left(R_{f}\right),$$ (17)

$$L=-\frac{1}{N}\overset{N}{\underset{i=1}{\sum }}Y_{i}^{\prime }\cdot log\left(Y_{i}\right),$$ (18)

where, in Equation 17, $Y^{\prime }$ is a m -length vector, the largest value index of $Y^{\prime }$ is the predicted label; Y is a one-hot vector representing the actual label. In Equation 18, L is the loss function, and N is the training image number.

## 4 Experimental configuration

### 4.1 Dataset description

Due to the confidentiality and sensitivity of medical data, as well as the high expertise and time costs required for medical image annotation, the use of public datasets has become a common and effective practice in the field of medical image analysis research. Public datasets, such as OCT (optical coherence tomography) image datasets, have been carefully collected and annotated by professional teams to ensure the quality and accuracy of the data. To evaluate our model’s effectiveness, we selected the two public OCT datasets: the OCT-2017 and the OCT-C8. The OCT-2017 dataset^1^ covers four types of retinal disease images: age-related wet maculopathy (CNV), diabetic macular edema (DME), age-related dry maculopathy (DRUSEN), and normal retinal images (NORMAL). The dataset comes from 4,686 patients with different eye diseases and contains a total of 84,484 images. There are 37,205 CNV images, 8,616 DRUSEN images, 11,348 DME images, and 26,315 NORMAL images in the training set. The testing set contains 1,000 images, with 250 each of various lesions and normal images, which are used to evaluate model performance. The OCT-C8 dataset^2^ contains a total of 24,000 images with eight categories. Each category has 2,300, 350, and 350 images for training, validation, and testing, respectively. The largest resolution of the OCT image is 384×496 , and the smallest resolution of the OCT image is 1536×496 .

In order to develop a unified model framework, we resize every OCT image into the same size: 512×512 pixels. The number of images in the original dataset is too different. During the training process, the accuracy of the category with the largest number will greatly affect the overall accuracy of the model. To solve this problem, this paper randomly selects an equal number from each category and determines the ratio of training, validating, and testing be 8:1:1. For the OCT-2017 dataset, we select 8,800 images for each category, including the 7,040 training images, 880 validating images, and 880 testing images. For the OCT-C8 dataset, we partitioned the dataset into the 8:1:1 ratio. The training, validating, and testing image numbers for each category are 2,400, 300, and 300, respectively. The datasets used for this study are summarized in Table 1. To accelerate the training speed and enhance the model’s ability to converge toward optimal weights, we normalize the image’s pixel values across its channels to a uniform range [0, 1]. This process ensures that the eigenvalues of the image data are within a comparable range, facilitating a more stable and efficient training process for neural networks. We also apply the image augmentation techniques (i.e., random shuffling, crop, and rotate) to enhance the generalization of the model’s performance.

**TABLE 1:** Experimental data details used in this study.

Dataset		AMD	CNV	CSR	DME	MH	Drusen	DR	Normal
OCT2017	Train	—	7,040	—	7,040	—	7,040	—	7,040
	Val	—	880	—	880	—	880	—	880
	Test	—	880	—	880	—	880	—	880
OCT-C8	Train	2,400	2,400	2,400	2,400	2,400	2,400	2,400	2,400
	Val	300	300	300	300	300	300	300	300
	Test	300	300	300	300	300	300	300	300

### 4.2 Model training details

In the Conv block, S=512 , and $C_{1}=4,C_{2}=8,C_{3}=C=16$ , there are L=3 retinal Mamba modules. Our model was trained using the TensorFlow framework on the Nvidia RTX4090 GPU. The Adam optimizer was selected for its adaptive learning rate adjustment capability, and the initial learning rate was set to 0.001 to promote rapid convergence while avoiding overfitting. The batch size is set at 64 to balance memory usage and training efficiency. The number of epochs was set to 150. After each round of dataset training, the model performance was evaluated through the validation set, and the learning rate or model structure was adjusted in time to optimize the results. During the training process, TensorBoard was used to monitor the changes in loss and accuracy to ensure that the training process was stable and effective. The trained model is evaluated on the testing set for comparison and analysis.

### 4.3 Evaluation metrics

In the multi-category classification task, we use the mean accuracy (mACC), mean sensitivity (mSEN), mean specificity (mSPE), mean precision (mPRE), mean F1-score (mF1), and overall accuracy (OACC). First, we compute the ACC, SEN, SPE, and PRE for each category and then average them for all the categories. During the evaluation, for each category, we treat it as a binary classification, where the positive label is itself and the negative label is the remaining categories. Therefore, TP represents the count of samples that are correctly identified as belonging to the positive category by the network’s predictions, matching their true-positive labels. FP denotes the number of samples that are incorrectly labeled as positive by the network’s predictions, despite their true labels being negative. TN stands for the count of samples that are accurately classified as negative by the network’s predictions, aligning with their genuine negative labels. FN signifies the number of samples that are erroneously classified as negative by the network, whereas their true labels are positive.

$$mACC=\frac{1}{m}\overset{m}{\underset{i=1}{\sum }}ACC_{i}=\frac{1}{m}\overset{m}{\underset{i=1}{\sum }}\frac{TP_{i}+TN_{i}}{N},$$ (19)

$$mSEN=\frac{1}{m}\overset{m}{\underset{i=1}{\sum }}SEN_{i}=\frac{1}{m}\overset{m}{\underset{i=1}{\sum }}\frac{TP_{i}}{TP_{i}+FN_{i}}.$$ (20)

$$mSPE=\frac{1}{m}\overset{m}{\underset{i=1}{\sum }}SPE_{i}=\frac{1}{m}\overset{m}{\underset{i=1}{\sum }}\frac{TN_{i}}{TN_{i}+FP_{i}}.$$ (21)

$$mPRE=\frac{1}{m}\overset{m}{\underset{i=1}{\sum }}PRE_{i}=\frac{1}{m}\overset{m}{\underset{i=1}{\sum }}\frac{TP_{i}}{TP_{i}+FP_{i}}.$$ (22)

$$mF1=\frac{1}{m}\overset{m}{\underset{i=1}{\sum }}F1_{i}=\frac{1}{m}\overset{m}{\underset{i=1}{\sum }}\frac{2\cdot PRE_{i}\cdot SEN_{i}}{PRE_{i}+SEN_{i}}.$$ (23)

where N is the testing image number and $ACC_{i}$ means the accuracy for the i -th category. Another OACC evaluates the overall performance for all categories. In the confusion matrix, we define TL as the diagonal of the matrix, and the OACC is expressed by

$$OACC=\frac{TL}{N}.$$ (24)

Equations 19–24 are used to evaluate the diagnosis performance of different methods on the ADNI and ABIDE datasets.

## 5 Results

### 5.1 Prediction results

**FIGURE 5:**
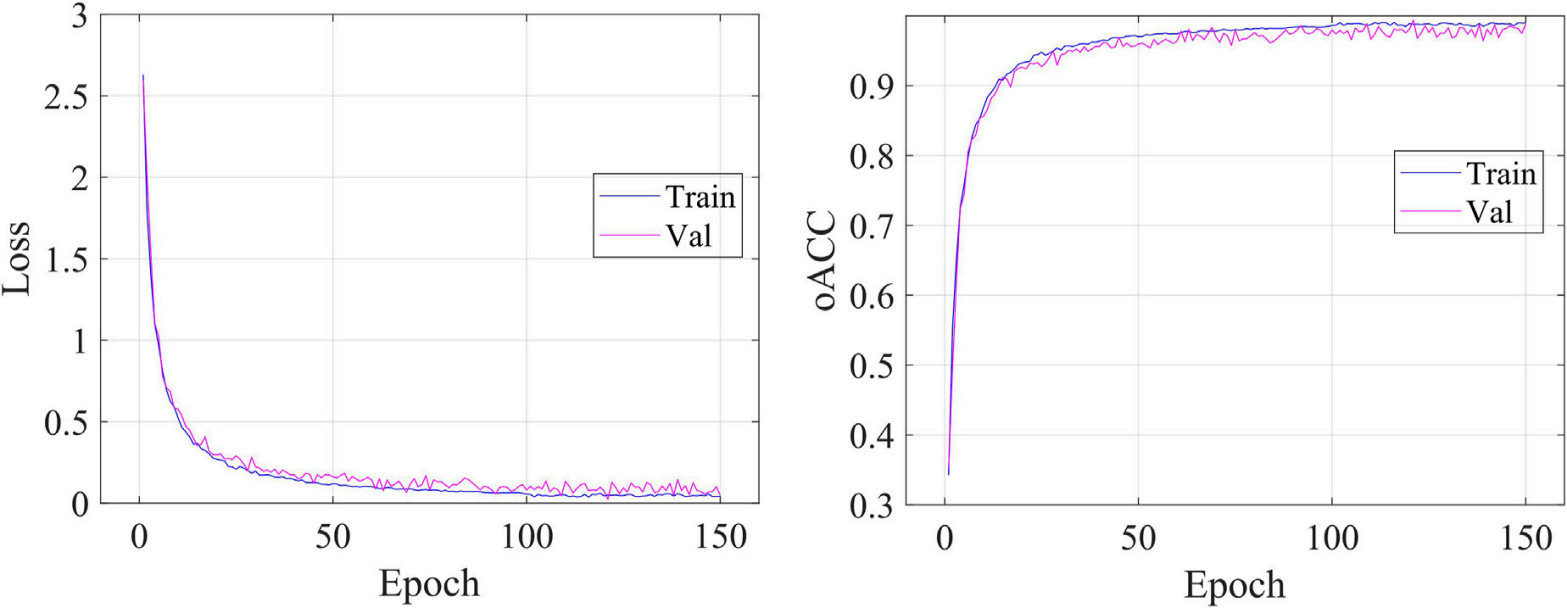
Visualization of objective loss and over accuracy during the model’s training.

**FIGURE 6:**
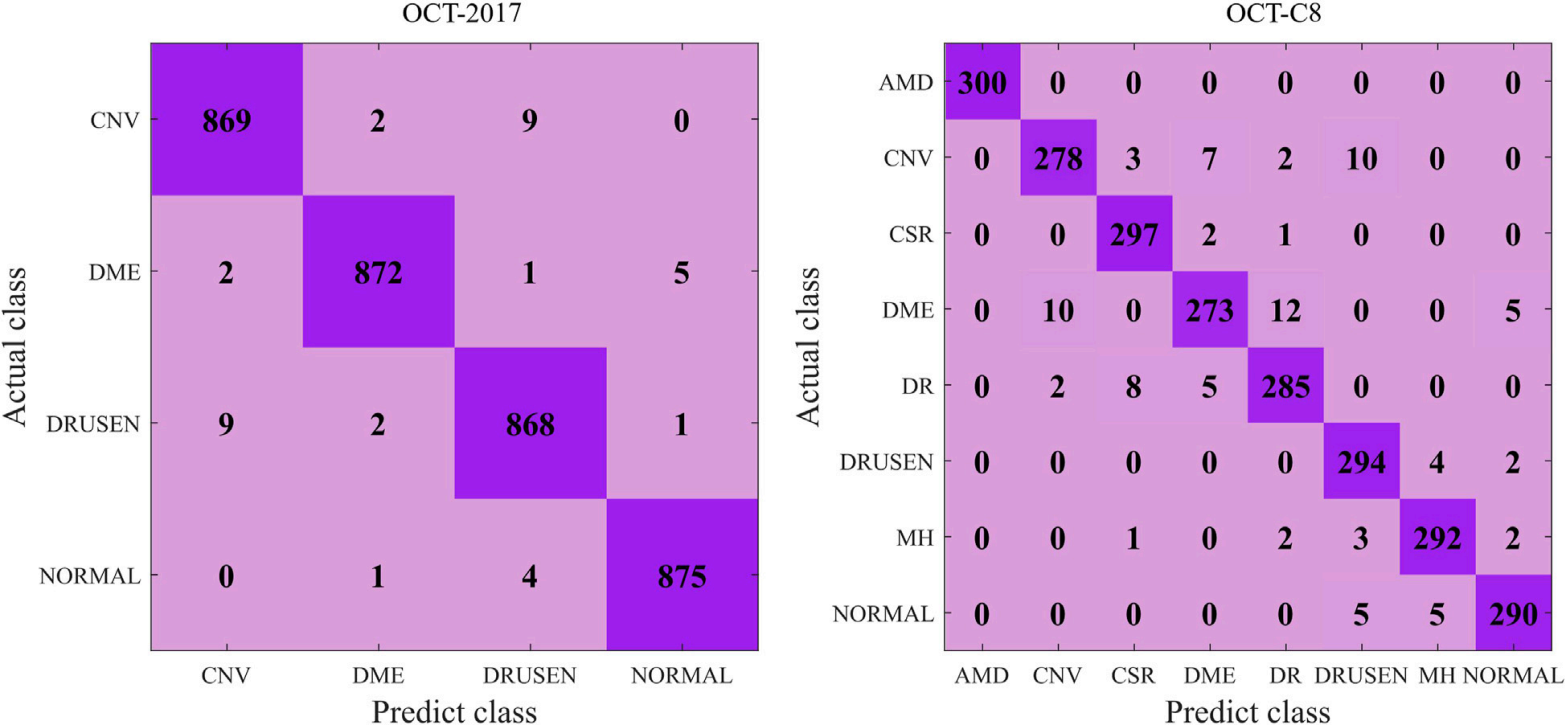
Confusion matrix of the predicted results on the OCT-2017 and OCT-C8 datasets using our model.

Figure 5 shows the details during the training. The left graph shows the curve of loss changing with epochs, and the right subfigure shows the curve of overall accuracy changing with the epochs. Both the training and validating losses show a stable trend. The little gap between them indicates that our model is a good fit model. The confusion matrix of the classification results is shown in Figure 6. Our model shows accurate classification performance on the OCT2017 dataset, with almost no errors in each category. In the OCT-C8 dataset, our model also performs well on most categories, except the CNV and DME categories. Table 2 shows the classification performance of the model on two different datasets (OCT2017 and OCT-C8). For each category, the ACC, SEN, PRE, F1, and SPE of each category are calculated according to the binary classification algorithm. For the OCT-2017 dataset, the average accuracy (mACC) and overall accuracy (oACC) of the model are 99.49% and 98.98%, respectively. For the OCT-C8 dataset, the overall accuracy of the model is 96.21%. Although the model achieved 100% of the indicators in the AMD category, the sensitivity in the CNV and DME classifications was relatively low (92.67% and 91.00%, respectively), resulting in a slight decrease in the F1 values of these categories. The results of these two datasets show that this model can maintain a high classification performance when dealing with tasks of multi-category classification.

**TABLE 2:** Detection results of our model on the two datasets. (%).

Dataset	Label	ACC	SEN	PRE	F1	SPE	oACC
OCT2017	CNV	99.38	98.75	98.75	98.75	99.58	98.98
	DME	99.63	99.09	99.43	99.26	99.81	
	DRUSEN	99.26	98.64	98.41	98.52	99.47	
	Normal	99.69	99.43	99.32	99.38	99.77	
	Average	99.49	98.98	98.98	98.98	99.66	
OCT-C8	AMD	100.00	100.00	100.00	100.00	100.00	96.21
	CNV	98.58	92.67	95.86	94.24	99.43	
	CSR	99.38	99.00	96.12	97.54	99.43	
	DME	98.29	91.00	95.12	93.02	99.33	
	DR	98.67	95.00	94.37	94.68	99.19	
	DRUSEN	99.00	98.00	94.23	96.08	99.14	
	MH	99.29	97.33	97.01	97.17	99.57	
	Normal	99.21	96.67	96.99	96.83	99.57	
	Average	99.05	96.21	96.21	96.19	99.46	

### 5.2 Comparative analysis

To demonstrate our model’s superiority, we select seven competing methods to test on our model and compare the classification performance. These methods include the baseline ResNet Talo et al. (2019), the CNN-based OctNet method Sunija et al. (2021), the ViT model Dosovitskiy et al. (2020), the Swin transformer model Liu et al. (2021), the CVM-Cervix model Liu et al. (2022), the CTransCNN model Wu et al. (2023), and the MedVit model Manzari et al. (2023). The last three hybrid models combine the CNN and transformer to conduct image classifications.

**FIGURE 7:**
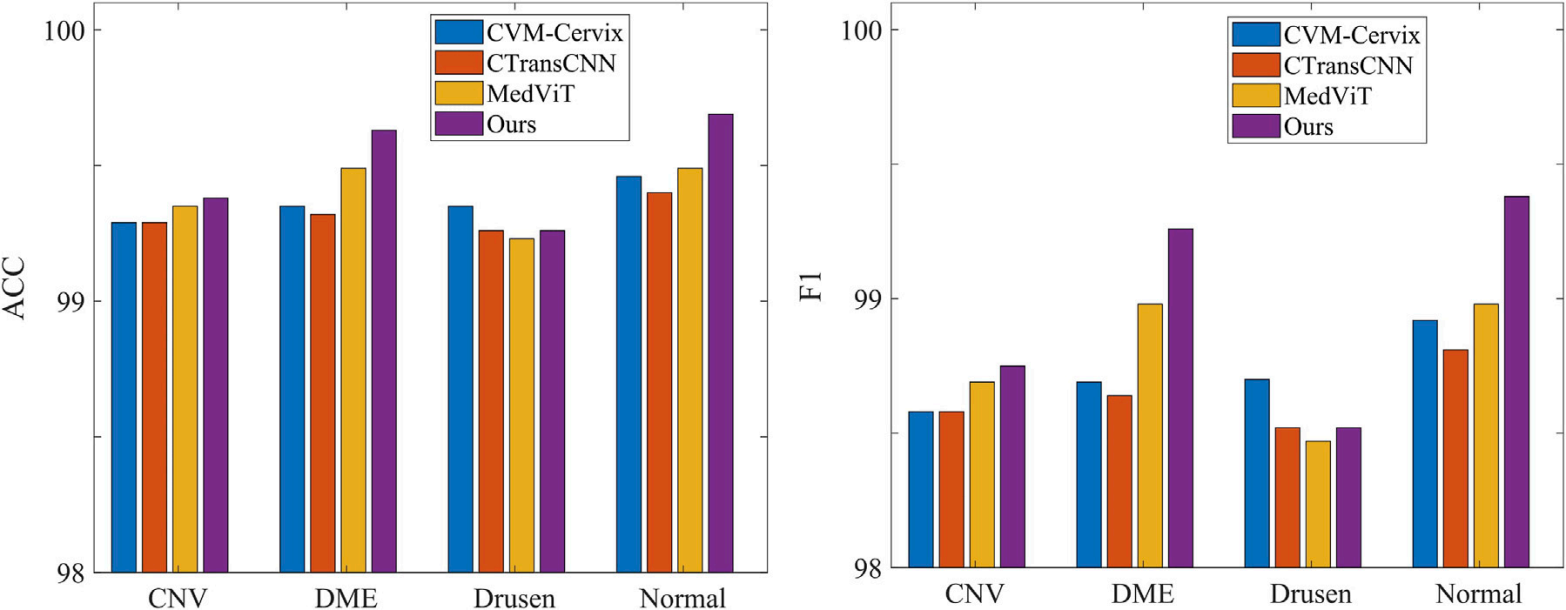
Comparison of different methods on the accuracy and F1 for each label (OCT-2017 dataset).

**FIGURE 8:**
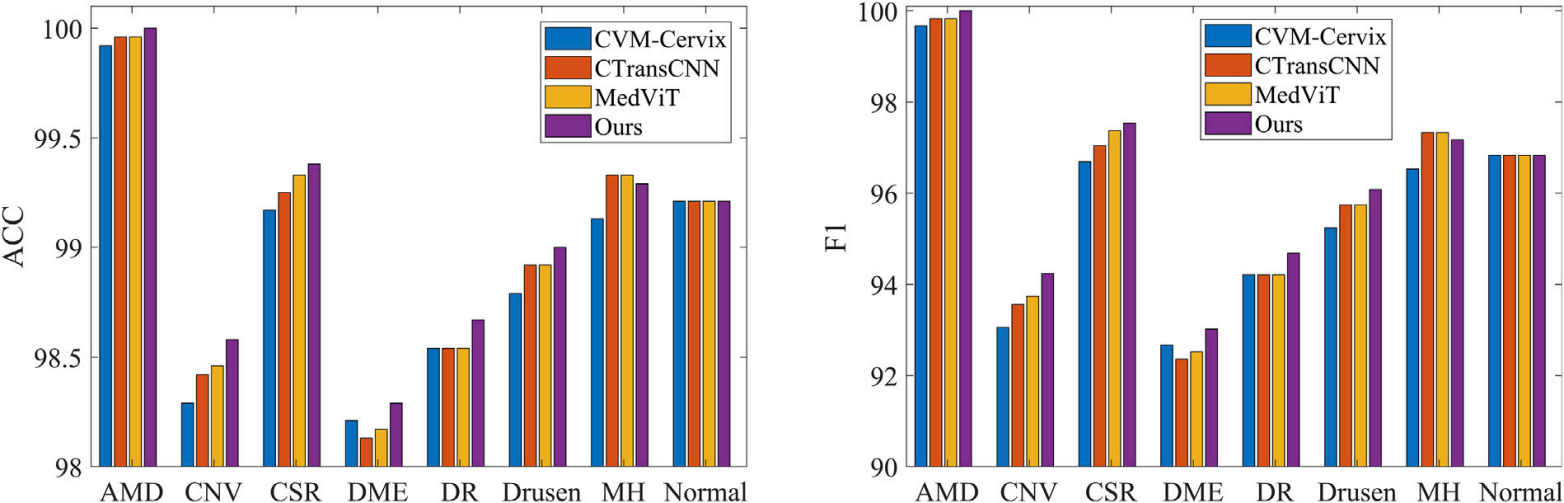
Comparison of different methods on the accuracy and F1 for each label (OCT-C8 dataset).

**FIGURE 9:**
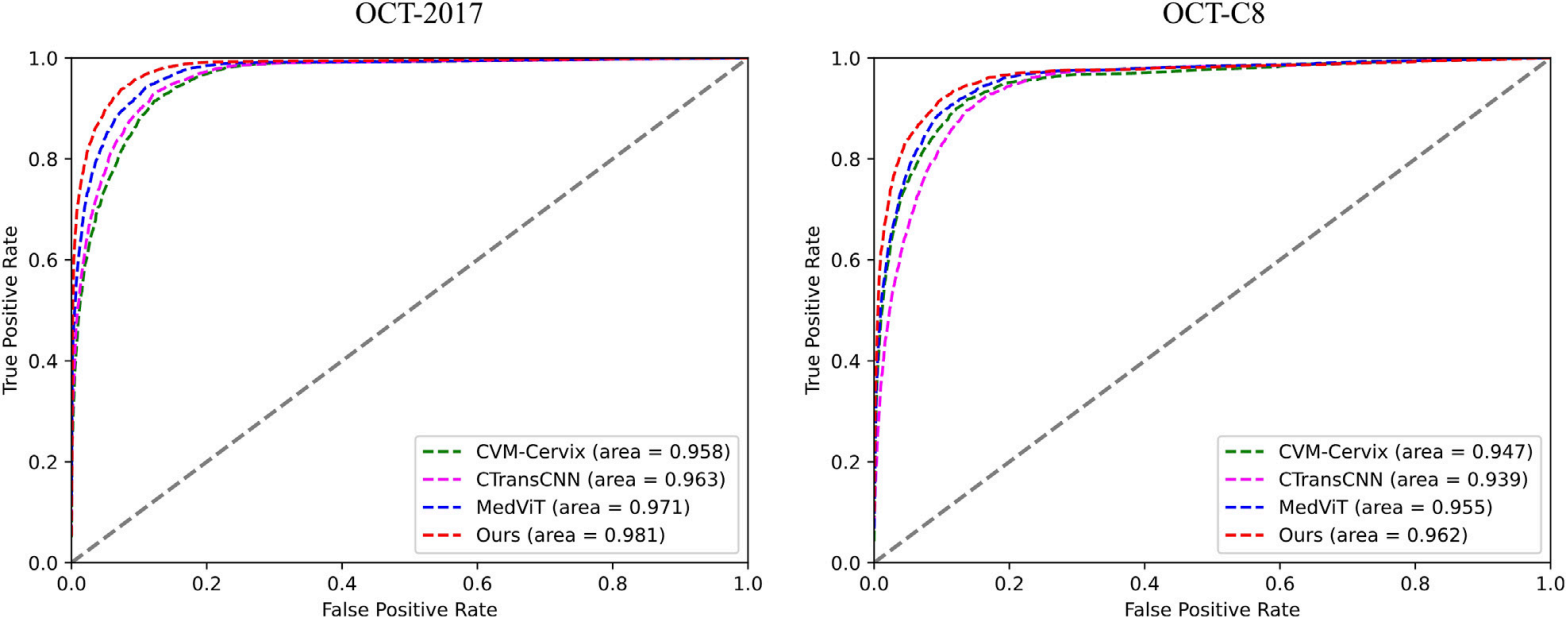
ROC comparison of the four different methods for both datasets.

**FIGURE 10:**
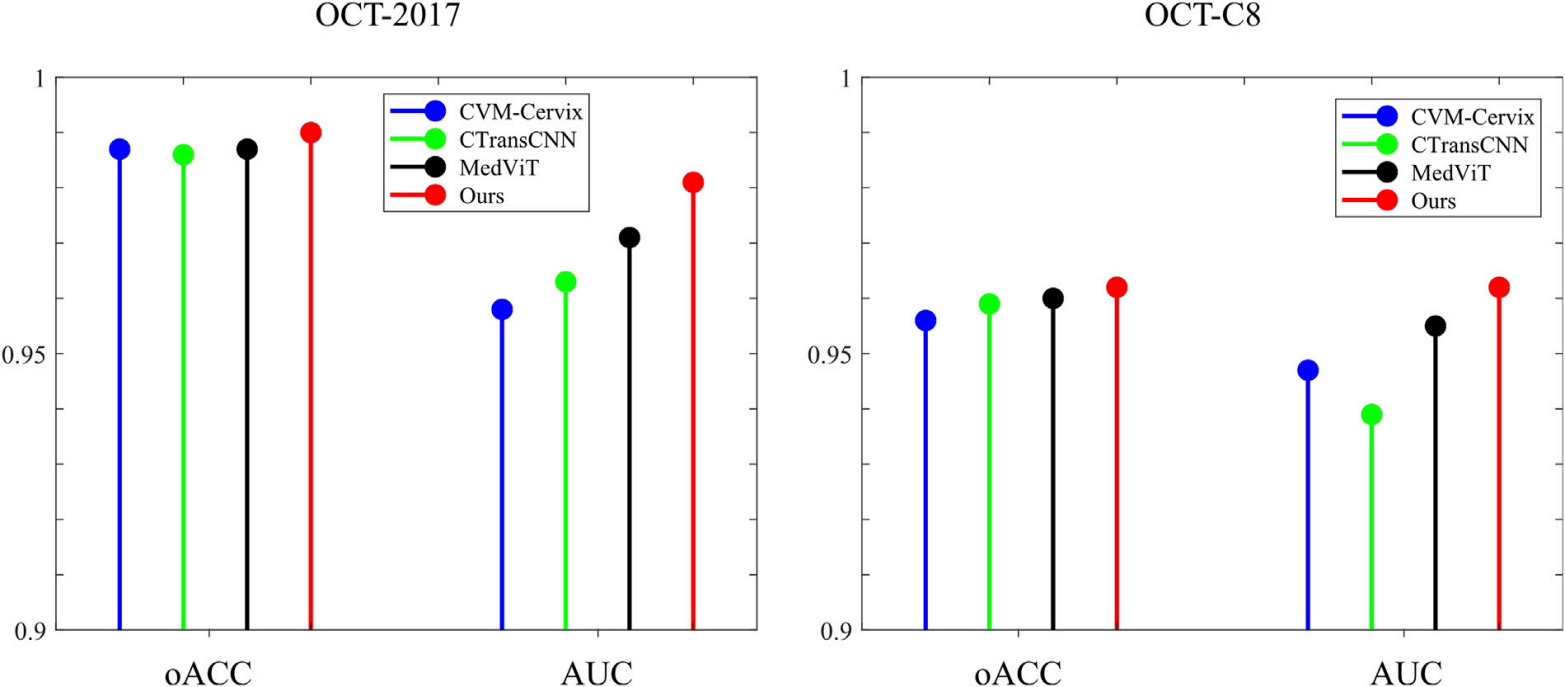
Stick diagram of the oACC and AUC on both datasets.

Table 3 demonstrates the comparison of the performance of different methods in multi-category classification tasks on the OCT2017 and OCT-C8 data sets. The evaluation indicators in the table include average accuracy (mACC), average sensitivity (mSEN), average precision (mPRE), average F1 value (mF1), average specificity (mSPE), and overall accuracy (oACC). On the OCT2017 dataset, our model performs best on all metrics, reaching an mACC value of 99.49% and an oACC value of 98.98%. On the OCT-C8 data set, our model also demonstrates strong generalization capabilities, outperforming other methods with an mACC of 99.05% and an oACC of 96.20%. Furthermore, we compare our model with the three hybrid models in terms of the ACC and F1 for each category. Figures 7, 8 show the classification performance of four methods (CVM-Cervix, CTransCNN, MedViT, and Ours) on the OCT-2017 dataset and OCT-C8 dataset, respectively. For the CNV category, our method slightly outperforms other methods in both ACC and F1 values, but the advantage is not obvious. For the DME category, our method significantly outperforms other methods, especially on the F1 value. For the Drusen category, both ACC and F1 values of our method are better than CVM-Cervix and CTransCNN, but slightly lower compared to MedViT. For the normal category, our method has significant advantages in both ACC and F1 values. We also compare the ROC of these four methods, and the results are shown in Figures 9, 10. Our model has the highest AUC value of 0.981 and 0.962 for OCT-2017 and OCT-C8, respectively. Our method has the best classification performance in overall accuracy and high AUC among these competing methods.

**TABLE 3:** Comparison of the multi-category classification using different methods. (%).

Dataset	Method	mACC	mSEN	mPRE	mF1	mSPE	oACC
OCT2017	ResNet50 Talo et al. (2019)	97.59	95.17	95.18	95.17	98.39	95.17
	OctNet Sunija et al. (2021)	98.37	96.73	96.74	96.73	98.91	96.73
	ViT Dosovitskiy et al. (2020)	98.93	97.87	97.87	97.87	99.29	97.87
	Swin Transformer Liu et al. (2021)	99.16	98.32	98.32	98.32	99.44	98.32
	CVM-Cervix Liu et al. (2022)	99.36	98.72	98.72	98.72	99.57	98.72
	CTransCNN Wu et al. (2023)	99.32	98.64	98.64	98.64	99.55	98.64
	MedViT Manzari et al. (2023)	99.39	98.78	98.78	98.78	99.59	98.78
	Ours	99.49	98.98	98.98	98.98	99.66	98.98
OCT-C8	ResNet50 Talo et al. (2019)	98.08	92.33	92.36	92.34	98.90	92.33
	OctNet Sunija et al. (2021)	98.32	93.29	93.31	93.30	99.04	93.29
	ViT Dosovitskiy et al. (2020)	98.47	93.88	93.88	93.87	99.13	93.88
	Swin Transformer Liu et al. (2021)	98.74	94.96	94.96	94.94	99.28	94.96
	CVM-Cervix Liu et al. (2022)	98.91	95.63	95.63	95.61	99.37	95.63
	CTransCNN Wu et al. (2023)	98.97	95.88	95.88	95.86	99.41	95.88
	MedViT Manzari et al. (2023)	98.99	95.96	95.96	95.95	99.42	95.96
	Ours	99.05	96.21	96.21	96.19	99.46	96.20

### 5.3 Ablation studies

To investigate the influence of different modules on the evaluation performance, we focus on the convolutional module (Conv), the multi-resolution (MR) strategy, the multi-path (MP) in the retinal Mamba, and the enhanced Mamba (EM). The MR removal means that we only keep the retinal Mamba in resolution-1. The MP removal means we remove the path-2, path-3, and path-4 in the retinal Mamba network. Removing EM means we delete the R-SiLU module in the enhanced Mamba.

Table 4 shows the impact of different modules (Conv, MR, MP, and EM) on the classification performance of our model. Specifically, the combination of all modules (Conv, MR, MP, and EM) performed best on both OCT2017 and OCT-C8 datasets. After removing the EM module, the classification performance shows an approximately 0.1 percent decrease. The Conv and MR modules both contribute to the improvement of our model’s classification performance. We further remove the Mamba-related modules (including MR, MP, and EM), and the oACC decreased by approximately 2.1 percentage and 1.2 percentage points on the OCT-2017 dataset and OCT-C8 dataset, respectively. This shows that each module plays an important role in the model, especially the Conv module and MR module, which are particularly critical to improving the overall performance. The lack of any module will lead to a decrease in the classification performance.

**TABLE 4:** Impact of different MRVM modules on the detection performance. (%).

Model	Conv	MR	MP	EM	OCT2017		OCT-C8	
					mACC	oACC	mACC	oACC
Our model	✗	✓	✓	✓	99.05	98.10	98.83	95.33
	✓	✗	✓	✓	99.16	98.32	98.93	95.71
	✓	✓	✗	✓	99.23	98.47	98.89	95.54
	✓	✓	✓	✗	99.32	98.64	98.94	95.75
	✓	✗	✗	✓	98.69	97.39	98.83	95.33
	✓	✗	✗	✗	98.44	96.88	98.75	95.00
	✓	✓	✓	✓	99.49	98.98	99.05	96.21

### 5.4 Discussion

**FIGURE 11:**
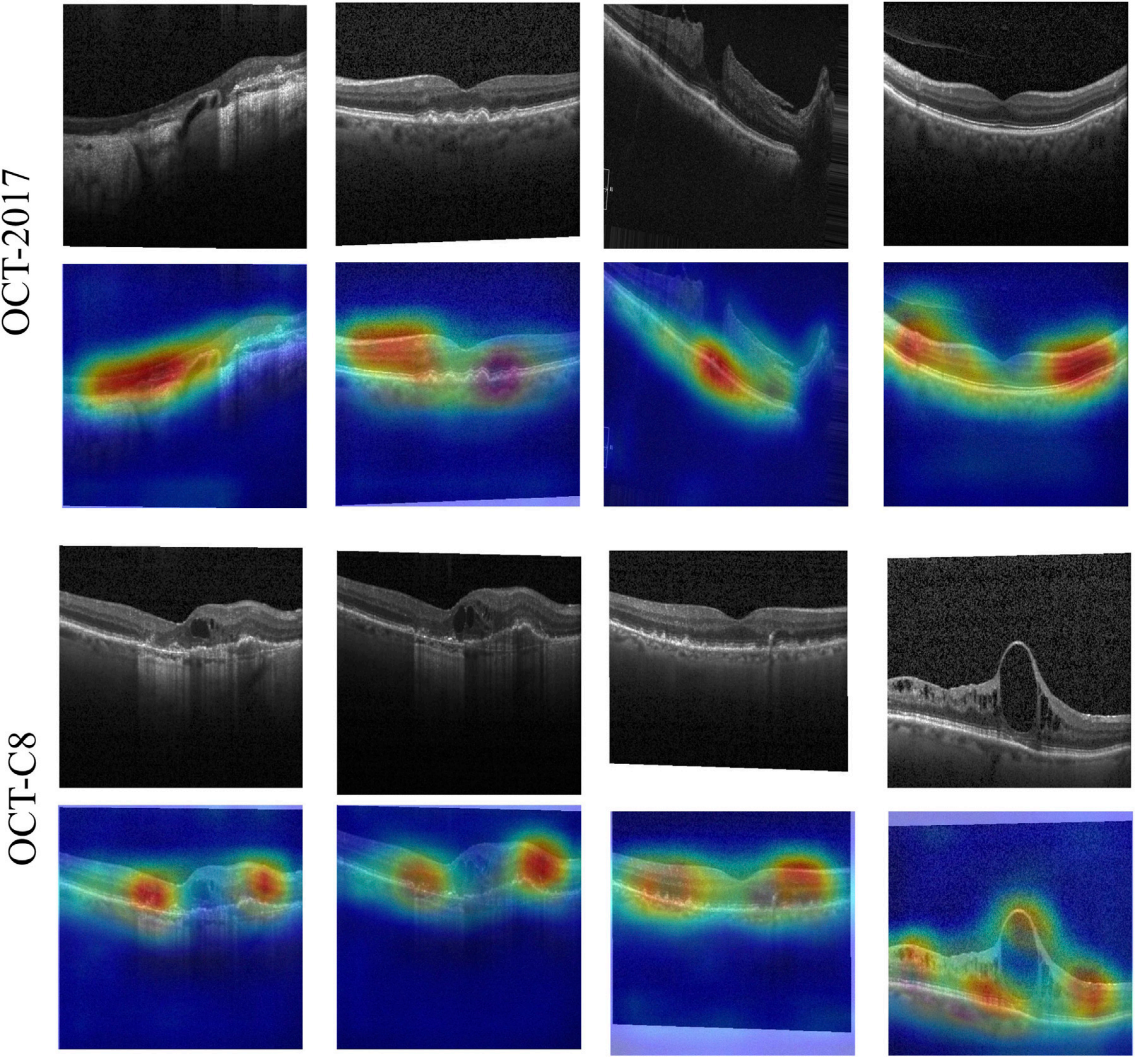
Activation heatmaps of different retinal OCT images using our model. The upper row is the raw OCT images, and the bottom row is the activation heatmaps using the Grad-CAM method.

Our model demonstrates good classification performance and generalization on two public datasets. Comparative analysis using different competing methods also shows our model’s superiority. The good performance of our model can be attributed to its great ability in feature extraction at multi-scales. Both global dependencies and local receptive fields can explore the underlying complex disease-related cues. The gradient-weighted class activation mapping (Grad-CAM) visualization can analyze and understand activation regions of different classes. We use it to show how our model captures the key cues in the retinal OCT image classification. As shown in Figure 11, the use of the Grad-CAM generates a heatmap with the size of the raw OCT image and shows the key areas in the OCT image that contribute most to the predicted label. To investigate our model’s robustness, we added a certain degree of noise to the original OCT images and followed the same training procedures. Table 5 shows the classification performance of our model under multiple noise levels. For the OCT2017 dataset, as the noise level increases from 0% to 10%, the mACC and oACC decrease from 99.49% and 99.98% to 99.19% and 98.38%, respectively. For the OCT-C8 dataset, the mACC and oACC decrease from 99.05% and 96.21% to 98.93% and 95.71%, respectively. Similarly, despite the slight performance degradation caused by noise, the model still maintains high accuracy and robustness under the influence of noise. Overall, the performance of the model under different noise conditions shows strong stability, especially under low-to-medium noise levels (1% and 5%); the classification performance only fluctuates slightly, indicating that the model has good resistance to noise.

**TABLE 5:** Impact of different levels of noise on the classification performance. (%).

Dataset	Noise level (%)	mACC	mSEN	mPRE	mF1	mSPE	oACC
OCT2017	0	99.49	98.98	98.98	98.98	99.66	99.98
	1	99.42	98.84	98.84	98.84	99.61	98.84
	5	99.35	98.69	98.69	98.69	99.56	98.69
	10	99.19	98.38	98.38	98.38	99.46	98.38
OCT-C8	0	99.05	96.21	96.21	96.19	99.46	96.21
	1	99.04	96.17	96.16	96.15	99.45	96.17
	5	99.00	96.00	96.00	95.98	99.43	96.00
	10	98.93	95.71	95.71	95.69	99.39	95.71

The main limitation of our model is the lack of multimodal retinal images. Single-modality retinal OCT images may not capture all pathological features of the retina. Single-modality retinal images can only provide information on one aspect but lack a comprehensive understanding of the global perspective. A single modality may not be able to fully assess the progression of the disease or other relevant pathological features. In the next study, we will add multimodal retinal images (i.e., fundus images) to more precisely detect retinal diseases.

## 6 Conclusion

This paper presents the multi-resolution visual Mamba (MRVM) model, designed to enhance OCT image classification performance by addressing long-range dependencies with linear computational complexity. The MRVM model first utilizes convolution operations to extract local features from OCT images and then leverages the retinal Mamba to capture global dependencies. By integrating multi-scale global features, the model not only improves classification accuracy but also boosts overall performance and robustness. A key innovation of the MRVM is its multi-directional selection mechanism, which enhances feature extraction by focusing on various directions to capture intricate, orientation-specific retinal patterns. Experimental results demonstrate that the MRVM model excels in distinguishing diverse retinopathy images, achieving a significant accuracy improvement over traditional methods—0.2 percentage points higher—with overall accuracies of 98.98% and 96.21% on the OCT2017 and OCT-C8 datasets, respectively. This advancement holds promise for automatic retinal disease diagnosis and could be valuable in clinical settings.

## Data Availability

The raw data supporting the conclusion of this article will be made available by the authors, without undue reservation.

## Footnotes

1
http://data.mendeley.com/datasets/rscbjbr9sj/2

2
http://kaggle.com/datasets/obulisainaren/retinal-oct-c8
